# Modulating functional amyloid formation via alternative splicing of the premelanosomal protein PMEL17

**DOI:** 10.1074/jbc.RA120.013012

**Published:** 2020-04-10

**Authors:** Dexter N. Dean, Jennifer C. Lee

**Affiliations:** Laboratory of Protein Conformation and Dynamics, Biochemistry and Biophysics Center, NHLBI, National Institutes of Health, Bethesda, Maryland 20892

**Keywords:** amyloid, tryptophan, electron microscopy (EM), protein aggregation, fluorescence, functional amyloid, melanosome, TEM, disaggregation, premelanosomal protein, PMEL17, melanin biosynthesis, alternative splicing

## Abstract

The premelanosomal protein (PMEL17) forms functional amyloid fibrils involved in melanin biosynthesis. Multiple PMEL17 isoforms are produced, two of which arise from excision of a cryptic intron within the amyloid-forming repeat (RPT) domain, leading to long (lRPT) and short (sRPT) isoforms with 10 and 7 imperfect repeats, respectively. Both lRPT and sRPT isoforms undergo similar pH-dependent mechanisms of amyloid formation and fibril dissolution. Here, using human PMEL17, we tested the hypothesis that the minor, but more aggregation-prone, sRPT facilitates amyloid formation of lRPT. We observed that cross-seeding by sRPT fibrils accelerates the rate of lRPT aggregation, resulting in propagation of an sRPT-like twisted fibril morphology, unlike the rodlike structure that lRPT normally adopts. This templating was specific, as the reversed reaction inhibited sRPT fibril formation. Despite displaying ultrastructural differences, self- and cross-seeded lRPT fibrils had a similar β-sheet structured core, revealed by Raman spectroscopy, limited-proteolysis, and fibril disaggregation experiments, suggesting the fibril twist is modulated by N-terminal residues outside the amyloid core. Interestingly, bioinformatics analysis of PMEL17 homologs from other mammals uncovered that long and short RPT isoforms are conserved among members of this phylogenetic group. Collectively, our results indicate that the short isoform of RPT serves as a “nucleator” of PMEL17 functional amyloid formation, mirroring how bacterial functional amyloids assemble during biofilm formation. Whereas bacteria regulate amyloid assembly by using individual genes within the same operon, we propose that the modulation of functional amyloid formation in higher organisms can be accomplished through alternative splicing.

## Introduction

Amyloids are a class of self-assembling proteins that form micron-length filaments with distinct cross β-sheet structure, where highly organized β-strands stack perpendicularly to the fibril growth axis (1). Although the aggregation of proteins into amyloid fibrils is traditionally associated with pathology (2), some proteins form amyloid fibrils that serve distinct biological functions (3). Such “functional amyloids” were first discovered as the principal component of extracellular biofilms formed by *Escherichia coli*, regulated by the expression of two *csg* (curli specific gene) operons (4, 5). Since then, functional amyloids have been discovered in all kingdoms of life, including humans (3, 6).

One example of a human functional amyloid is the premelanosomal protein (PMEL17, herein referred to as PMEL), which promotes melanin biosynthesis in melanocytes by forming functional amyloid fibers that serve as a scaffold for melanin polymerization (7). This occurs within the melanosome, an acidic, membranous organelle analogous to the lysosome that goes through four distinct stages of maturation (8). During stage I, PMEL is processed into a small membrane subunit and a large luminal domain, termed Mα. Mα undergoes further processing during stage II of melanosome maturation to yield the domain(s) responsible for amyloid formation (9, 10). Melanin then begins to polymerize on fibrils during stage III and fully coats the melanosome upon full maturation at stage IV (8). During melanosome maturation, the intralumenal pH increases from acidic (pH ∼4) in early stages to near-neutral pH upon full maturation (11). Previous work from our lab and others have shown that the repeat (RPT) domain, constituting residues 315–444, forms amyloid fibrils *in vitro* under acidic conditions similar to those found in the early stages of melanosome maturation (9, 10, 12–21). Exposure of preformed RPT fibrils to cytosolic conditions (pH ≥7) leads to rapid dissolution *in vitro*, which is proposed to protect against cytotoxicity *in vivo* should fibrils escape the melanosome (9, 10, 17). Additionally, six of nine missense *PMEL* mutations identified in heritable pigmentary glaucoma localize to the RPT domain and disrupt fibril formation *in vivo* (22).

**Figure 1.**
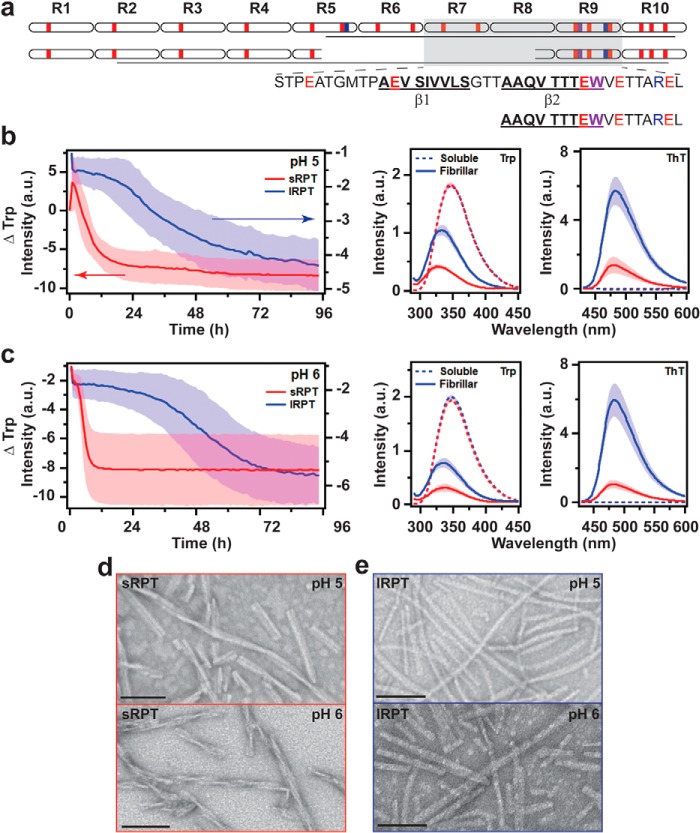
**Comparison of sRPT and lRPT fibril formation as a function of pH.** *a*, schematic representation of the primary sequence of lRPT (*top*) and sRPT (*bottom*). Acidic, basic, and tryptophan residues are colored in *red*, *blue*, and *purple*, respectively. *Lines* represent the protease-resistant region defined in this work. Amino acids defining the putative β-strands in lRPT fibrils, as defined previously (16), are also shown. *b* and *c*, aggregation kinetics of 30 μm lRPT (*blue*) and sRPT (*red*) at pH 5 (*b*) and 6 (*c*) under constant linear shaking (3 mm) at 37 °C. Trp and ThT fluorescence were collected of soluble (*dashed*) and fibrillar (*solid*) RPT before and after aggregation reactions, respectively. Spectra are normalized for respective concentration. *Solid line* and *shaded* area represent the mean and S.D. from three independent experiments, respectively. *d* and *e*, representative TEM images of sRPT (*d*) and lRPT (*e*) fibrils. *Scale bars* represent 100 nm.

The most abundant isoform of PMEL contains a RPT domain consisting of 10 imperfect repeats of 13 amino acids each (schematically shown in Fig. 1*a*, *top*). RPT is rich in acidic residues (highlighted in *red*), the protonation of which at low pH leads to charge neutralization and drives amyloid formation (16). In particular, those found in the C-terminal repeats 7–9 are the most important, as this region forms the amyloid core as characterized by limited-protease digestion (16) and solid-state NMR spectroscopy (15). Two putative β-strands have been proposed within this region (16). Excision of a cryptic intron within the RPT domain leads to a PMEL isoform lacking R6 and R7, as well as portions of R5 and R8 (Fig. 1*a*) (23). The two PMEL isoforms can be expressed independently, leading to a scenario wherein both could be present in the melanosome (23). In the case of MNT-1 human melanoma cells, the smaller PMEL isoform represents ∼10% of the total PMEL mRNA (23). The shorter RPT isoform (sRPT)^2^ shares the β2 motif (Fig. 1*a*) with the longer RPT isoform (lRPT), and both have a highly pH-dependent aggregation/disaggregation *in vitro* (17, 21). For a more detailed review of PMEL17 functional amyloid formation, we refer the readers to recently published review articles (9, 10).

Given that both RPT isoforms form amyloid fibrils *in vitro*, this led us to question the purpose of the shorter RPT isoform. In bacteria, functional amyloid assembly occurs through a nucleated polymerization mechanism, where a minor subunit protein rapidly forms an amyloid template that nucleates the assembly of the major subunit protein (24–27). The two amyloidogenic proteins are expressed as separate genes within the same operon, and the “nucleator” protein shares sequence homology with the major subunit protein. Therefore, we hypothesize that alternative splicing within RPT, which produces a minor isoform (sRPT), serves to facilitate the amyloid formation of the major isoform (lRPT). To examine this, we performed cross-seeding experiments, where preformed fibril seeds were added to soluble protein to measure the effect on aggregation kinetics, and the resulting fibrillar materials were characterized by Trp fluorescence, Raman spectroscopy, as well as transmission EM (TEM). We find that sRPT fibrils accelerate lRPT aggregation, similar to how CsgB nucleates CsgA functional amyloid formation in *E. coli* (28). Based on bioinformatic analysis, alternatively spliced RPT domains in PMEL homologs exist in other mammals, suggesting a conserved modulation mechanism of PMEL amyloid formation by short and long isoforms. Overall, this work offers insights into RPT amyloid interplay and suggests that there are parallels between bacterial and human functional amyloids in using different proteins or isoforms to regulate fibril formation.

## Results and discussion

### Comparison between sRPT and lRPT aggregation kinetics

First, lRPT and sRPT aggregation kinetics were compared at solution conditions mimicking middle (pH 5) and late (pH 6) stages of melanosome maturation (11). Reactions were monitored at 37 °C under constant agitation (3 mm linear shaking) by intrinsic tryptophan (Trp) fluorescence. The single Trp residue in the C terminus (Fig. 1*a*) resides in the protease-resistant cores of both lRPT and sRPT and is a sensitive probe of fibril formation (13, 16, 17). Upon fibril formation, the Trp emission exhibits a concomitant shift to higher energy (blue-shift) and decreased intensity, indicating the change in its local environment polarity as well as π-π stacking as the monomers assemble (13). As previously reported, both aggregations of sRPT and lRPT were highly pH-dependent with lag times increasing with pH (Fig. 1, *b* and *c*). Further, sRPT exhibited faster kinetics compared with lRPT. For comparison, the time it takes to reach one half of the maximum change in Trp fluorescence (*t*_½_) indicated that sRPT aggregated 10- to 70-fold faster than lRPT (Table S1). The biphasic kinetics observed at pH 5 for sRPT is attributable to fibril formation and maturation, based on prior work (17).

### Molecular differences between sRPT and lRPT fibrils

To identify differences between soluble and aggregated RPT at different pH, shifts in the Trp emission maximum (λ_max_) were compared (Fig. 1, *b* and *c*). For the soluble protein, λ_max_ of ∼348 nm was measured regardless of the protein or pH. Upon aggregation, varying blue-shifts were observed (Δλ_max_ ∼10–19 nm), indicating that the Trp side chain had transitioned from a water-exposed to a more hydrophobic local environment (Table S1). Additionally, nearly identical amide-I peaks centered at 1665 cm^−1^, characteristic for β-sheet secondary structure (29), were observed for all samples (Fig. S1). Interestingly, although the expected thioflavin-T (ThT) fluorescence enhancement was verified for all samples, normalizing for the respective fibril concentration revealed that ThT fluorescence intensity was higher for lRPT compared with that of sRPT fibrils (Fig. 1, *b* and *c*), suggesting distinct fibril structures. Ultrastructural differences were also visualized in negatively stained TEM images. A twisted filament architecture was observed for sRPT fibrils (Fig. 1*d*), whereas lRPT formed straight (rodlike) fibrils (Fig. 1*e*).

To reveal the underlying molecular differences within the fibrils, limited proteolysis and mass spectrometry (MS) were used to identify protease-resistant regions. Fibrils were incubated at 37 °C overnight (∼15 h) in the presence of proteinase-K (PK), followed by SDS-PAGE and LC-MS analyses (Fig. S2). Upon incubation with PK, full-length bands disappeared and gave rise to a smaller band migrating at ∼5 kDa (Fig. S2). Using LC-MS, peptide fragments were mapped (Fig. S2 and Table S2). For lRPT, the PK-resistant amyloid core constituted residues 373–442, whereas a core spanning nearly the entire length of the sequence containing residues 332–398 was observed for sRPT (Fig. 1*a*), clearly showing that sRPT and lRPT fibrils are different. Collectively, these data show that not only does sRPT aggregate faster than lRPT, but also that distinct fibril morphologies are adopted by each protein.

### sRPT fibrils increase the rate of lRPT aggregation

**Figure 2.**
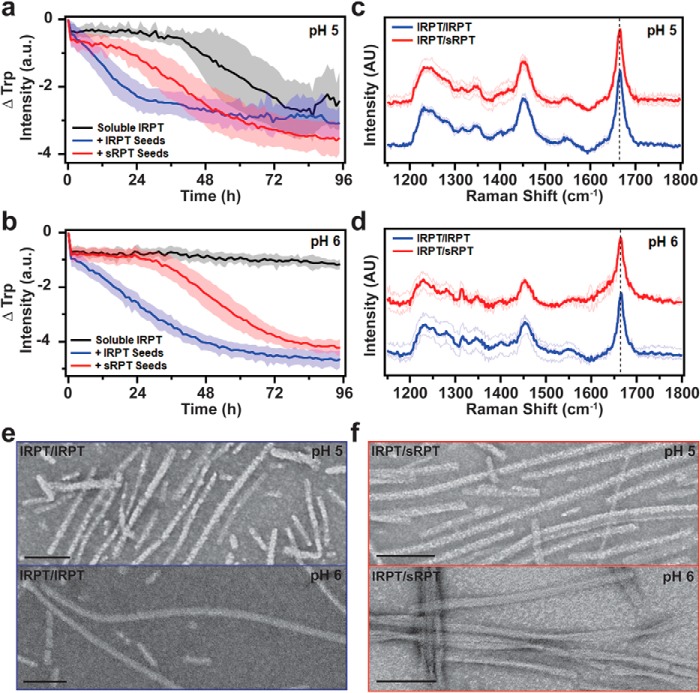
**Cross-seeding reactions of lRPT by preformed sRPT fibrils.** *a* and *b*, aggregation of 30 μm lRPT in the absence (*black*, unseeded) and in the presence of 3 μm preformed lRPT (*blue*, self-seeding) or sRPT (*red*, cross-seeding) fibrils at pH 5 and 6. Reactions were monitored under constant linear shaking (6 mm) at 37 °C. *Solid line* and *shaded area* represent the mean and S.D., respectively, from ≥5 replicates. See Fig. S7 for additional data set. *c* and *d*, self- (lRPT/lRPT, *blue*) and cross-seeded (lRPT/sRPT, *red*) fibrils, formed at pH 5 and 6, were analyzed by Raman spectroscopy. Spectra were collected at multiple spatial locations (*thin lines*), which were then averaged (*bold line*). The vertical *dashed line* represents 1665 cm^−1^, to which data were normalized. Cross-seeded spectra are offset for clarity. *e* and *f*, representative TEM images of lRPT/lRPT and lRPT/sRPT fibrils. *Scale bars* represent 100 nm.

To explore how sRPT influences lRPT fibril formation, cross-seeding reactions were performed by adding preformed sRPT fibrils (seeds) to soluble lRPT and monitoring Trp fluorescence (Fig. 2, *a* and *b*). Seeds were added at a 10% molar ratio, which mimics melanosomal ratios of RPT isoforms found *in vivo* (23). In these experiments, agitation was decreased (6 mm linear shaking) to minimize *de novo* fibril formation, for which lRPT did not aggregate in the absence of seeds within the given time frame at pH 6 (Fig. 2*b*, *black*). Upon the addition of sRPT seeds (cross-seeding, *red curves*), lRPT aggregation was stimulated at both pH 5 and 6 (Fig. 2, *a* and *b*), albeit with reduced potency compared with the addition of lRPT seeds (self-seeding, *blue curves*), which eliminated the lag phase of aggregation altogether.

A similar blue-shift of Trp fluorescence and increase in ThT fluorescence was seen for self-seeded (lRPT/lRPT, *blue*) and cross-seeded (lRPT/sRPT, *red*) fibrils (Fig. S3, *a* and *b*). Additional Raman (Fig. 2, *c* and *d*) and TEM (Fig. 2, *e* and *f*) characterization indicated little difference between secondary structural and microscopic features for lRPT/lRPT and lRPT/sRPT fibrils at pH 5. We reason that, because *de novo* aggregation is still possible under these conditions, there is competition between soluble lRPT-soluble lRPT and soluble lRPT-fibrillar sRPT interactions as there is an excess of soluble lRPT in solution. In comparison, a difference in fibril morphology was visualized by TEM for lRPT/sRPT fibrils at pH 6 (Fig. 2*f*), despite having a similar Raman fingerprint (Fig. 2*d*). Although a rodlike morphology is the predominant species for unseeded (Fig. 1*e*) and self-seeded lRPT fibrils (Fig. 2*e*), cross-seeded lRPT/sRPT fibrils at pH 6 displayed a twisted protofilament architecture (Fig. 2*f*), similar to that observed for unseeded sRPT (Fig. 1*d*). Here, because lRPT does not aggregate by itself at pH 6, the sRPT seeds are able to have a greater impact on fibril morphology. However, we note that upon closer inspection of the numerous TEM images taken, some twisted protofilaments were observed in lRPT/lRPT fibrils, although they were sparse in number and not the predominant morphology (Fig. S4). Despite the appearance of twisted fibrils, the same PK-resistant core was determined for lRPT/lRPT and lRPT/sRPT fibrils (Fig. S5). Together with the Raman data, this suggests that regions outside the amyloid core may be responsible for defining the twist architecture. These results of sRPT fibrils increasing the rate of lRPT aggregation are analogous to how CsgB nucleates CsgA curli fibrils in *E. coli* (28). Furthermore, our data suggest that lRPT can adopt an sRPT-like fold at pH 6 by sRPT-fibril templated growth.

### lRPT fibrils inhibit sRPT fibril maturation

**Figure 3.**
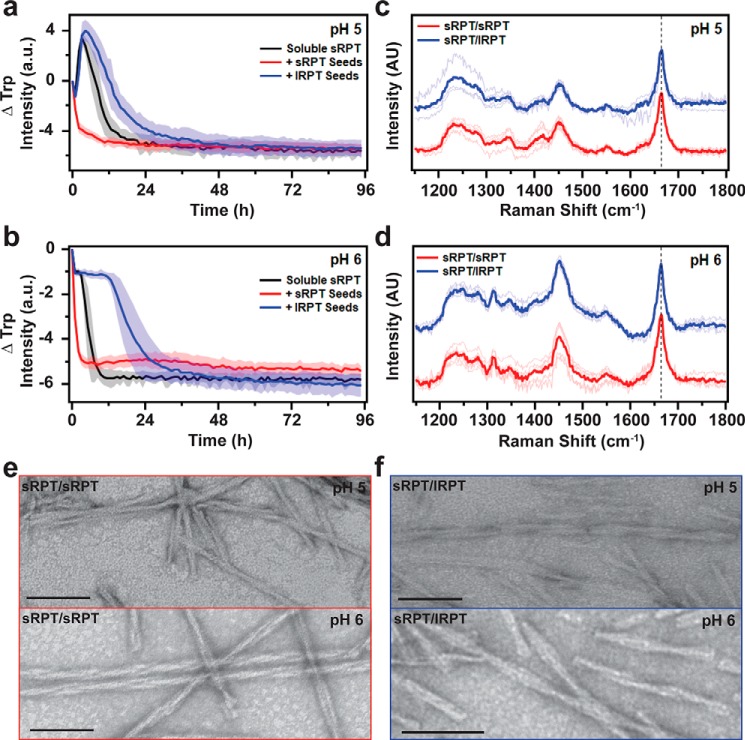
**Cross-seeding reactions of sRPT by preformed lRPT fibrils.** *a* and *b*, aggregation of 30 μm sRPT in the absence (*black*, unseeded) and in the presence of 3 μm preformed sRPT (*red*, self-seeding) or lRPT (*blue*, cross-seeding) fibrils at pH 5 and 6. Reactions were monitored under constant linear shaking (6 mm) at 37 °C. *Solid line* and *shaded area* represent the mean and S.D., respectively, from ≥5 replicates. See Fig. S7 for additional dataset. *c* and *d*, self- (sRPT/sRPT, *red*) and cross-seeded (sRPT/lRPT, *blue*) fibrils, formed at pH 5 and 6, were analyzed by Raman spectroscopy. Spectra were collected at multiple spatial locations (*thin lines*), which were then averaged (*bold line*). The vertical *dashed line* represents 1665 cm^−1^, to which data were normalized. Cross-seeded spectra are offset for clarity. *e* and *f*, representative TEM images of sRPT/sRPT and sRPT/lRPT fibrils. *Scale bars* represent 100 nm.

Next, we investigated the reverse reaction, *i.e.* whether lRPT fibrils influenced sRPT aggregation. At pH 5, addition of lRPT seeds to soluble sRPT (*blue curve*) did not change the initial increase in Trp intensity compared with unseeded sRPT (*black curve*); however, it protracted the subsequent maturation phase (Fig. 3*a*) in a seed concentration–dependent manner (Fig. S6). Interestingly, this inhibition saturated at 20% (molar ratio) seeding, indicating that sRPT monomers bind to lRPT fibrils in a 5:1 stoichiometric ratio (Fig. S6). In contrast, self-seeding (*red curve*) completely abolished this initial phase and accelerated the second phase, revealing that the presence of sRPT fibril seeds leads directly to mature fibril formation (17). The inhibitory effect of lRPT fibrils became more pronounced at pH 6, increasing the *t*_½_ time by 12 h (Fig. 3*b*; unseeded = 4 h, cross-seeded = 16 h). Although slight variations in Trp kinetics were observed from experiment to experiment, consistent overall trends were observed (Fig. S7).

Similar λ_max_ values were observed for self- (sRPT/sRPT, *red*) and cross-seeded (lRPT/sRPT, *blue*) fibrils at pH 5 and 6 (Fig. S3, *c* and *d*), although the Trp intensity was noticeably increased for sRPT/lRPT fibrils at pH 5, because of the presence of immature fibrils, which have higher Trp intensity. ThT fluorescence enhancement was similar for self- and cross-seeded sRPT fibrils at pH 6, but again, differences were seen for cross-seeded sRPT/lRPT fibrils at pH 5 (Fig. S3*c*), which may be because of contributions from the lRPT fibril seeds. Secondary structures and protease-resistant cores of sRPT/sRPT and sRPT/lRPT fibrils were largely analogous (Fig. 3, *c* and *d*, and Fig. S5). Correspondingly, no differences in the fibril architecture between sRPT/sRPT and sRPT/lRPT fibrils could be discerned by TEM, with both displaying a twisted protofilament morphology (Fig. 3, *e* and *f*). From these data, it is revealed that although lRPT seeds can inhibit the kinetics of sRPT fibril maturation, the overall fibril structure remains largely unaffected. It is evident that although lRPT is able to template from sRPT seeds, the reverse does not occur.

The specificity with which this cross-seeding occurs could be explained by the fact that the lRPT protein contains all the amino acids found in sRPT and given that these are imperfect repeat sequences, it is possible to shift the register of residues to accommodate fibril growth. We propose that the β2 region present in both lRPT and sRPT facilitates lRPT templating onto sRPT seeds, although surface-mediated secondary nucleation (30) cannot be ruled out. On the other hand, sRPT lacks some of the critical residues which are needed to template onto the lRPT seeds, particularly the β1 motif (Fig. 1*a*). Although future experiments are needed to define the specific molecular interactions between sRPT and lRPT, our results are clear that sRPT fibrils stimulates lRPT aggregation.

### lRPT fibrils disaggregate faster than sRPT fibrils

**Figure 4.**
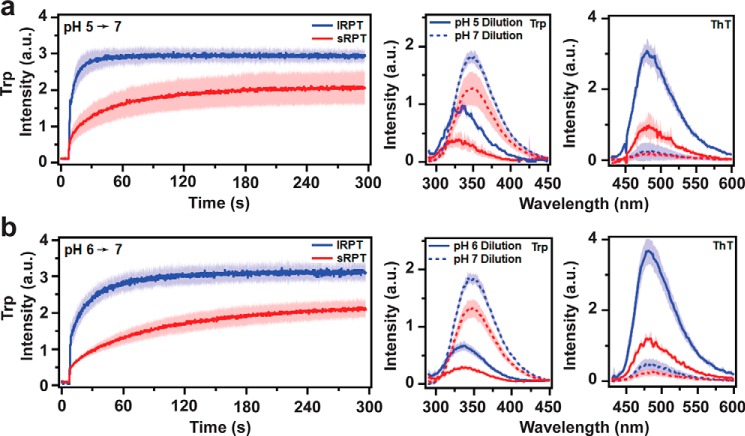
**Comparison of disaggregation of lRPT and sRPT fibrils at pH 7.** *a* and *b*, lRPT (*blue*) and sRPT (*red*) fibrils (10 μm) formed at pH 5 (*a*), or 6 (*b*) were diluted 6-fold into pH 7 buffer and Trp emission was monitored under constant stirring at 25 °C. Trp and ThT fluorescence were collected after self-dilution (*solid lines*) or dilution into pH 7 buffer (*dashed lines*). *Bold line* and *shaded* area represent the mean and S.D. from three independent experiments, respectively.

One unique feature of RPT fibrils is that they rapidly disaggregate upon deprotonation of acidic residues at neutral pH (14, 16, 17), which has been suggested as a means to protect against cytotoxicity should PMEL fibrils escape the acidic environment of the melanosome. Here, we compared the rates of disaggregation between lRPT and sRPT fibrils and studied how cross-seeding impacts this process. First, the disaggregation of unseeded lRPT and sRPT fibrils were evaluated by Trp fluorescence as they were diluted 6-fold into a cuvette containing pH 7 buffer (Fig. 4). The initial sharp increase in Trp fluorescence observed at ∼10 s represents the moment when RPT fibrils were added, after which changes in Trp intensity report on conformational changes in the protein as a result of disaggregation (Fig. 4). Upon self-dilution (*i.e.* fibrils formed at pH 5 into pH 5 buffer), no changes in Trp intensity were observed after the initial mixing event, indicating dilution alone did not result in disaggregation (Fig. S8). However, upon dilution into pH 7 buffer, all samples displayed an exponential increase in Trp fluorescence, indicative of disaggregation (Fig. 4). Further, final Trp spectral profiles at the end of the pH 7 reactions (*dashed lines*) showed a red-shift in the λ_max_ to 348 nm, suggesting the residue was in a polar (water-exposed) environment similar to that observed prior to aggregation. A significant reduction in ThT fluorescence was also observed upon dilution into pH 7 buffer, altogether supporting that fibril disassembly had occurred.

**Figure 5.**
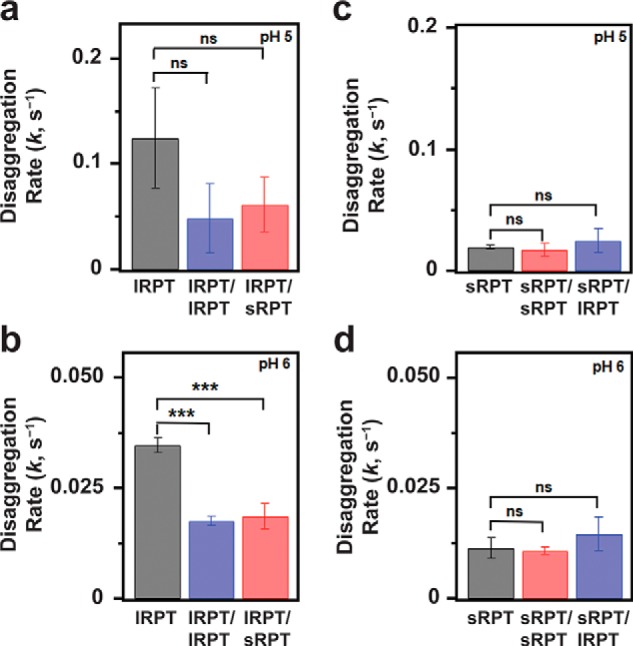
**Disaggregation of self- and cross-seeded RPT fibrils.** *a–d*, disaggregation rate constants, *k*, extracted from single-exponential fits of kinetic curves shown in Fig. 4 for unseeded lRPT (*a* and *b*) or sRPT (*c* and *d*) and Fig. S9 for self- and cross-seeded fibrils. *p* values were calculated from three independent experiments using unpaired *t* tests (GraphPad), where *ns* and *** represent *p* > 0.05 and *p* ≤ 0.001, respectively.

To quantify the rate of disaggregation, kinetics data were fit to an exponential growth equation to determine the rate constant (*k*) (Fig. 5). Unseeded sRPT fibrils displayed rate constants 6- and 3-fold lower than unseeded lRPT fibrils at pH 5 and 6, respectively (Fig. 5, *p* ≤ 0.02), which is correlated to its enhanced aggregation propensity compared with lRPT. However, although biphasic kinetics were observed for sRPT aggregation at pH 5, disaggregation is well-described by a single exponential curve. This suggests that aggregation/disaggregation for sRPT is more nuanced than lRPT and is not simply the reverse of aggregation (*i.e.* perhaps the involvement of different protonation sites).

### Self- and cross-seeding of lRPT results in decreased rates of disaggregation

Next, the disaggregation of self- and cross-seeded fibrils was compared (Fig. 5 and Fig. S9). Similar to that of the unseeded samples, an exponential increase in Trp fluorescence was observed upon dilution into pH 7 buffer, which was also accompanied by a red-shift in the λ_max_ of Trp fluorescence and a reduction in ThT fluorescence (Fig. S9). However, the extracted rate constants of both lRPT/lRPT and lRPT/sRPT fibrils were reduced by half compared with the unseeded sample (Fig. 5, *a* and *b*). This suggests that lRPT fibrils formed in the presence of either lRPT or sRPT seeds resulted in a more stable fibril structure, especially at pH 6. It should be pointed out that although lRPT self- and cross-seeded aggregation kinetics differed (Fig. 2, *a* and *b*), their disaggregation kinetics were nearly identical (Fig. S9, *a* and *b*).

The implication here is that although self- and cross-seeded lRPT adopted different ultrastructural fibril morphologies (rod *versus* twisted), they had no measurable impact on fibril sensitivity to pH and fibril dissolution. This is in agreement with the Raman (Fig. 2*d*) and PK (Fig. S5) data, which were similar for lRPT/lRPT and lRPT/sRPT fibrils. Although we initially expected that some molecular differences would be observed through either spectroscopic or biochemical analyses, it is evident that no discernable features between self- *versus* cross-seed lRPT fibrils were revealed using these methods, which probed the structured amyloid core. Based on these results, we attribute the ultrastructural differences seen by TEM to regions (*i.e.* N-terminal residues) outside the amyloid core. This explanation is supported by recent structures of full-length and C-terminally truncated α-synuclein, where different fibril twist architectures (periodicities ranging from 121 to 64 nm) are adopted by highly similar core structures (31). Notably, the α-synuclein fibrils are more twisted as the unstructured C terminus is removed and the twisted morphology can be propagated to full length using the shorter construct. Perhaps, in the case of RPT, fibril twist is also influenced by sterics imposed by regions outside the amyloid core. Obviously, more work is needed to elucidate the structural differences by high-resolution methods such as solid-state NMR or cryo-EM.

Unlike that observed above for lRPT, no reduction in the disaggregation rate constant was observed for either sRPT/sRPT or sRPT/lRPT fibrils compared with unseeded sRPT (Fig. 5, *c* and *d*). This implies that even though self-seeding of sRPT induced immediate fibril growth (no lag phase), this did not translate to increased fibril stability (*i.e.* slower disaggregation kinetics), likely because of the highly amyloidogenic nature of sRPT. Additionally, a marginal increase in the disaggregation rate constant for sRPT/lRPT fibrils was observed compared with sRPT/sRPT, suggesting that inhibition of fibril maturation (by lRPT fibrils) has only a modest effect on fibril stability (Fig. 5, *c* and *d*).

### Long and short RPT isoforms are found in PMEL homologs from other mammals

**Figure 6.**
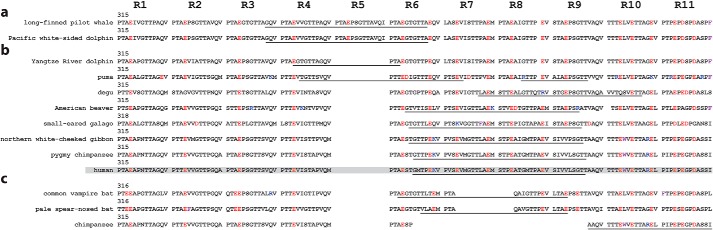
**Short and long RPT isoforms from mammalian PMEL homologs.** *a–c*, one-letter amino acid sequences of the RPT domains from mammalian PMEL homologs, with acidic, basic, and aromatic residues shown in *red*, *blue*, and *purple*, respectively. Species were grouped as being longer (*a*), similar in length (*b*), or shorter in length (*c*) compared with human RPT (shaded in *gray*). *Underlined regions* represent the residues which are absent in the short isoform for each species.

The results described above indicate a parallel between what we observed for RPT and the well-established mechanism of curli amyloid formation in *E. coli*, where CsgB nucleates the polymerization of CsgA during biofilm formation (28). A similar mechanism of functional amyloid formation is also suggested in other bacteria, such as the nucleation of FapC by its homolog FapB in *Pseudomonas* (24) as well as TapA, which has been shown to nucleate TasA amyloid formation in *Bacillus subtilis* (27). This led us to explore if PMEL homologs from other mammals also contain long and short isoforms of RPT, which would give additional evidence that this could be a conserved mechanism of PMEL amyloid formation. Using the National Center for Biotechnology Information (NCBI) Gene dataset, annotated genes for mammalian PMEL homologs having more than one reported isoform were aligned using NCBI's constraint-based multiple alignment tool (COBALT) to see if they differed in the RPT region. Of these, 20 were found to have short and long isoforms of RPT (Table S3), where a selected few are shown in Fig. 6. Overall, the RPT region was found to be highly conserved, particularly the location of acidic residues (colored *red*) within each repeat. However, the overall number of repeats and the areas which are spliced out in the short isoforms (*underlined regions*) varied. Although most PMEL homologs contain 10 repeats similar to humans (Fig. 6*b*), some were longer (Fig. 6*a*) or shorter (Fig. 6*c*) than humans. Strikingly, the long RPT isoform found in chimpanzees was nearly identical to the short RPT isoform found in humans. Further, chimpanzees have a short RPT isoform that lacks the C-terminal repeats 9–11. Overall, this reveals a conserved process of alternative splicing within the RPT domain of mammalian PMEL homologs, the shorter of which may be responsible for nucleating functional amyloid formation similar to what has been described here for human sRPT.

## Conclusions

We have shown here that a minor isoform of the human PMEL RPT domain (sRPT) can cross-seed and accelerate the aggregation of the major PMEL RPT isoform (lRPT) *in vitro*, which mirrors functional amyloid assembly in bacteria. To our knowledge, this is the first demonstration of nucleated polymerization via cross-propagation among human functional amyloids. The cross-seeded fibrils display morphological features unique to sRPT, yet the amyloid core is unaffected, which suggests that N-terminal residues outside of the amyloid core are responsible for defining the macroscopic fibril architecture.

We hypothesize that cross-seeding may be conserved among other eukaryotic functional amyloid systems, as has been previously proposed for cystatin-related epididymal spermatogenic (CRES) proteins which form a functional amyloid matrix in the epididymal lumen of mice (32–34). Although bacteria achieve nucleated polymerization by clustering minor and major amyloid subunits as individual genes within the same operon, we provide an example where the same mechanism is accomplished by alternative splicing. Although it is not clear how prevalent alternative splicing is for eukaryotic functional amyloids, the discovery of alternatively spliced RPT isoforms in other mammals suggests it may be a conserved mechanism of PMEL functional amyloid formation.

## Experimental procedures

### Materials

Unless otherwise noted, all chemicals and reagents/equipment were procured from Sigma-Aldrich and VWR International, respectively. UltraPure^TM^ guanidine hydrochloride (GuHCl), NuPAGE^TM^ 4–12%, Bis-Tris Protein Gels, NuPAGE^TM^ LDS sample buffer (4×), SimplyBlue^TM^ SafeStain, and proteinase-K were purchased from Invitrogen. The Precision Plus Protein^TM^ dual color protein standard was purchased from Bio-Rad.

### Expression and purification of RPT

Plasmids encoding either lRPT or sRPT constructs with a C-terminal hexa-histidine tag were transformed into *E. coli* BL21(DE3) competent cells (Fisher Scientific) and expressed by the NHLBI Protein Expression Facility as described before (18). Cell pellets (∼10 g/purification) were then resuspended in 50 ml of lysis buffer (6 m GuHCl, 100 mm NaCl, 100 mm Na_2_HPO_4_, 20 mm imidazole, pH 7.4) and sonicated (on ice) for 60 s (50% duty cycle, output control = 5) using a 3-mm tapered microtip attached to a Branson Sonifier 450. After 2 min of rest (on ice), a second round of sonication was performed followed by overnight incubation (4 °C) on a Roto-Mini Plus rotating mixer to ensure complete lysis. Cell debris was then pelleted by centrifugation at 20,000 rpm (47,808 rcf) for 30 min at 4 °C. The lysate was then loaded onto a pre-equilibrated HisPrep^TM^ FF 16/10 column using an ÄKTA pure chromatography system (GE Healthcare). After washing out unbound material, RPT was eluted using an increasing concentration gradient of imidazole. Fractions corresponding to RPT were then pooled and dialyzed (4 °C) for at least 3 h against 4 liters of dialysis buffer (20 mm Tris, 1 mm ethylenediaminetetraacetic acid, pH 8.0) using a 3.5-kDa molecular weight cut-off Spectra/Por^®^3 membrane. After three rounds of dialysis, the protein was collected, aliquoted, and stored at −80 °C. The purity of the protein was confirmed to be >90% using SDS-PAGE and LC-MS.

### Aggregation reactions

Prior to exchanging lRPT or sRPT stocks into the desired buffer, any preformed aggregates were removed by filtering the protein through a 100-kDa molecular weight cut-off Amicon^®^ Ultracel membrane (EMD Millipore). The protein was then exchanged into prechilled pH 5 (20 mm sodium acetate, 100 mm NaCl) or pH 6 (20 mm MES, 100 mm NaCl) buffer using a Sephadex G-25 PD-10 gravity column (GE Healthcare). All buffers were sterilized using a 0.2 μm polyethersulfone membrane (EMD Millipore). The concentration of protein was determined by measuring the absorbance at 280 nm (ϵ = 5500 M^−1^ cm^−1^) on a Cary 300 series UV-visible spectrometer (Agilent Technologies). To generate fibril seeds, the protein was diluted to 30 μm and aliquoted (70 μl) into a black 384-well polypropylene flat bottom plate (Greiner Bio-One) and sealed using a MicroAmp optical adhesive film (Thermo Fisher Scientific). To facilitate aggregation, a presterilized 2-mm borosilicate glass bead was added to each well. Aggregation kinetics were monitored using intrinsic Trp fluorescence on a SPARK Multimode Microplate reader (Tecan) maintained at 37 °C. Measurements were collected every hour from the top using excitation and emission wavelengths of 280 and 350 nm, respectively. Between each time point, the plate was kept under constant linear shaking (3 mm). The initial sharp decrease in Trp intensity observed from 0 to 1 h is attributed to temperature equilibration and not thought to represent the aggregation of RPT. To better mimic conditions in the melanosome, preformed fibrils were not sonicated prior to initiating seeding experiments, which is often done to prepare fragmented filaments to enhance growth. Fibrils (3 μm) were added to 30 μm soluble protein and aggregation was monitored as described above, with the exception that reactions were agitated at 6 mm linear shaking. Data were processed by subtracting all time points for a particular well by its initial value at *t*_0_. Multiple wells (≥5) of the same sample were averaged and plotted along with 1 S.D. using IgorPro 7.08 software (WaveMetrics).

### Quantifying RPT fibrils

After aggregation reactions had gone to completion, aliquots from the 384-well plate were combined into a sterile microcentrifuge tube (Eppendorf) and centrifuged at 17,000 rcf for 30 min to pellet the insoluble fibrils. After removing the soluble material, fibrils were resuspended in fresh buffer supplemented with 0.01% sodium azide to prevent microbial growth. Fibrils were quantified by mixing an aliquot 1:1 with 8 m GuHCl and collecting the absorbance at 280 nm using a NanoDrop-1000 Spectrophotometer (Thermo Fisher Scientific).

### Fluorescence spectroscopy

Intrinsic Trp and extrinsic ThT fluorescence measurements were collected using a Fluorolog FL-3 instrument (Horiba Scientific) as described previously (17). Briefly, samples of 30 μm soluble or 10 μm fibrillar protein were made and Trp fluorescence was first collected by monitoring the emission from 290 to 450 nm while exciting at 280 nm. Then, ThT was added to the cuvette at a final concentration of 10 μm. After a 60-s equilibration period, fluorescence was measured from 430 to 600 nm while exciting at 415 nm. All spectra had been subtracted by buffer background and normalized for protein concentration. To determine the λ_max_ for Trp fluorescence, data were fit to a Gaussian distribution using the built-in function in Igor Pro 7.08 software (WaveMetrics).

### Disaggregation reactions

Disaggregation kinetics were monitored using a Fluorolog FL-3 instrument (Horiba Scientific) operating in kinetics mode as described previously (17). Briefly, RPT fibrils (80 μl of 10 μm supplemented with 10 μm ThT) were added to a 10-mm quartz cuvette containing 420 μl of pH 7 (20 mm sodium phosphate, 100 mm NaCl) buffer under constant stirring. Trp emission was continuously monitored at 350 nm with 280 nm excitation. Immediately after collecting kinetics data, Trp and ThT spectral scans were collected as described above. To determine the rate of reactions (*k*), data were fit to an exponential equation using the built-in function in Igor Pro 7.08 software (WaveMetrics).

### Transmission EM

One day prior to collecting images, grids were prepared by depositing 5 μl of fibrils (30–50 μm) onto a 400-mesh copper grid with a formvar/carbon film (Electron Microscopy Sciences) and allowing it to adhere for 60 s. After wicking excess sample away using Whatman filter paper, 5 μl of 2% (w/v) uranyl acetate was added and immediately wicked away. Grids were dried in a desiccator overnight prior to imaging on a JEOL JEM 1200 EXII microscope (NHLBI EM Core) equipped with an XR-60 digital camera (Advanced Microscopy Techniques) operating at 80 kV. Images are representative of several samples, each imaged at multiple grid squares using a magnification of 40,000×.

### Raman spectroscopy

Raman spectroscopy was collected on a home-built instrument (35). First, fibril samples were centrifuged at 17,000 rcf for 30 min. After removing the supernatant, the fibril pellet was resuspended in fresh buffer (without sodium azide) to achieve a concentration of ∼100 μm. A 20-μl droplet of the fibril sample was then deposited onto a Nunc LabTek chamber (no. 1 coverglass). Multiple spectra from varying spatial locations were collected, with each spectrum representing 30 accumulations (15-s integration time). Data were analyzed using LabSpec 6 software (Horiba Scientific) as previously reported (17). For comparison, intensities for all data were normalized to the amide-I band at 1665 cm^−1^.

### Limited-protease digestions

Fibrillar RPT samples (30 μm formed at pH 6) were incubated under constant agitation (600 rpm, VWR Mini-Micro 980140 shaker) in the absence or presence of 0.3 μg/ml proteinase-K at 37 °C. After 8 h (seeded fibrils) or 15 h (unseeded fibrils), aliquots of the sample were analyzed by SDS-PAGE by adding denaturing sample buffer to a concentration of 1× and heating on a 100 °C heat block for 10 min. After heating, samples were loaded onto a NuPAGE^TM^ 4–12%, Bis-Tris polyacrylamide gel and separated using a PowerEase^®^ 500 (Invitrogen) power supply operating at 200 V. To estimate molecular weights, Precision Plus^TM^ dual color protein standards were ran in parallel. Bands were visualized using SimplyBlue^TM^ SafeStain following the manufacture's protocol and imaged on a Typhoon imaging scanner using ImageQuant software (GE Healthcare). Samples were also analyzed by LC-MS using an Agilent 6224 electrospray ionization TOF (ESI-TOF) LC-MS instrument (NHLBI Biochemistry Core). The digestion reaction was mixed with 8 m GuHCl in a 1:2 (v/v) ratio, which was then diluted 1 volume with a solution of 5% acetonitrile (AcN)/0.05% (v/v) trifluoroacetic acid (TFA) in a LC-MS vial. Samples were then loaded onto a Zorbax StableBond 300 C18 column (Agilent Technologies) pre-equilibrated in 99% H_2_O/0.05% TFA (v/v) and eluted with a linear gradient of 99% AcN/0.05% TFA. Masses were extracted from individual peaks in the total ion chromatogram and deconvoluted using MassHunter software (Agilent Technologies) to identify peptide fragments. Theoretical masses of peptide fragments were calculated using the ProtParam tool from the ExPASy Bioinformatics Resource Portal (RRID: SCR_018087).

### Bioinformatic analysis of PMEL homologs

Mammalian PMEL homologs were identified using the “orthologs” function on the NCBI website (RRID: SCR_006472). First, genes were sorted to remove those in which only one isoform was available. The remaining PMEL homologs were analyzed individually using COBALT to determine whether the reported isoforms differed in the RPT region, which was considered to begin approximately at residue 315 with the PT*X*E motif. Of the 20 that displayed a long and short RPT domain (Table S3), they were next sorted by length compared with human PMEL RPT and aligned manually.

### Data availability

Datasets for all *in vitro* experiments described here will be freely provided upon request (Jennifer C. Lee, leej4@nhlbi.nih.gov). Gene accession numbers for PMEL homologs shown in Fig. 6 can be found in Table S3 and analyzed using the NCBI website (RRID: SCR_006472). All other data are contained within this article or the supporting information.

## Author contributions

D. N. D. and J. C. L. conceptualization; D. N. D. data curation; D. N. D. formal analysis; D. N. D. writing-original draft; J. C. L. supervision; J. C. L. writing-review and editing.

## Supplementary Material

Supporting Information

## Footnotes

2 The abbreviations used are:

sRPT: short repeat domain
lRPT: long repeat domain
rcf: relative centrifugal force
TEM: transmission EM
ThT: thioflavin-T.
