# Supplementary material for: Modulating functional amyloid formation via alternative splicing of the premelanosomal protein PMEL17
Source: J Biol Chem. 2020 Apr 10;295(21):7544–53. doi: 10.1074/jbc.RA120.013012 (PMC7247297; doi:10.1074/jbc.RA120.013012)
Supplement: Supporting Information [file supp_295_21_7544__index.html]

Modulating functional amyloid formation via alternative splicing of the premelanosomal protein PMEL17 — Amyloid Formation of PMEL17 isoforms — Modulating functional amyloid formation via alternative splicing of the premelanosomal protein PMEL17 — Amyloid formation of PMEL17 isoforms — Supporting Information 

# Modulating functional amyloid formation via alternative splicing of the premelanosomal protein PMEL17

## Supporting Information

- Supporting Information (to be published online) - Supporting Information Figures and Tables
