# Supplementary material for: Modulating functional amyloid formation via alternative splicing of the premelanosomal protein PMEL17
Source: J Biol Chem. 2020 Apr 10;295(21):7544–53. doi: 10.1074/jbc.RA120.013012 (PMC7247297; doi:10.1074/jbc.RA120.013012)
Supplement: Supporting Information [file supp_RA120.013012_158788_3_supp_507260_q8fvd3.pdf]

Supporting Information for:

Modulating Functional Amyloid Formation via Alternative Splicing of Pmel17

Dexter N. Dean and Jennifer C. Lee*

The Laboratory of Protein Conformation and Dynamics, Biochemistry and Biophysics Center, National Heart, Lung, and Blood Institute, National Institutes of Health, Bethesda, MD 20892, USA

*To whom correspondence should be addressed. Email: leej4@nhlbi.nih.gov

Table S1. Average $t_{1/2}$ and λ_{max} values determined for data in Figure 1

pH	$t_{1/2}$ (h)		λ_{max} (nm)	
	lRPT	sRPT	lRPT	sRPT
**5**	35	0.5	334	329
**6**	50	5.3	336	338

*For sRPT at pH 5, the initial increase in Trp fluorescence was used to determine the $t_{1/2}$.

Table S2. RPT fragments identified by LC-MS

Fragment	Theoretical Mass (Da)	Observed Mass (Da)		
		Unseeded lRPT	lRPT/lRPT	lRPT/sRPT
Full-length	13914.4	13914.6	13914.5	13914.6
N'-442	12891.3	12891.4	12891.4	12891.4
332-C'	12247.6	-	12247.6	12247.6
332-442	11224.5	-	11224.7	11224.7
371-C'	8427.4	8427.6	8427.6	8427.6
373-C'	8239.2	8239.3	8239.4	8239
371-442	7404.3	7404.3	7404.6	7404.2
373-442	7216.1	7216	7216.1	7216
393-442	5126.6	-	5126.6	5126.6
		Unseeded sRPT	sRPT/sRPT	sRPT/lRPT
Full-length	9694.5	9694.7	9694.7	9694.7
N'-400	8671.4	8671.3	8671.4	8671.4
332-C'	8027.7	8027.8	8027.9	8027.8
332-400	7004.7	7004.8	7004.8	7004.8
332-398	6846.5	6846.7	6846.6	6846.7

Table S3. List of Pmel homologs having both long and short RPT isoforms

species	gene accession number	
	long isoform	short isoform
human	NP_001186983.1	NP_001307050.1
water buffalo	XP_025139495.1	XP_025139498.1
great roundleaf bat	XP_019524620.1	XP_019524621.1
sea otter	XP_022378685.1	XP_022378684.1
prairie vole	XP_005371224.1	XP_026633811.1
Bolivian squirrel monkey	XP_010348249.1	XP_010348250.1
pale spear-nosed bat	XP_028387979.1	XP_028387981.1
common vampire bat	XP_024432074.1	XP_024432077.1
Philippine tarsier	XP_008062211.1	XP_021571200.1
degu	XP_023563148.1	XP_023563150.1
chimpanzee	XP_009423447.2	XP_009423449.2
Yangtze River dolphin	XP_007467815.1	XP_007467816.1
Arctic ground squirrel	XP_026254104.1	XP_026254106.1
small-eared galago	XP_012661050.1	XP_023369387.1
American beaver	XP_020038469.1	XP_020038472.1
puma	XP_025782524.1	XP_025782525.1
Pacific white-sided dolphin	XP_026962390.1	XP_026962391.1
long-finned pilot whale	XP_030722545.1	XP_030722546.1
pygmy chimpanzee	XP_008976891.1	XP_008976893.1
northern white-cheeked gibbon	XP_030678330.1	XP_030678331.1

Figure S1

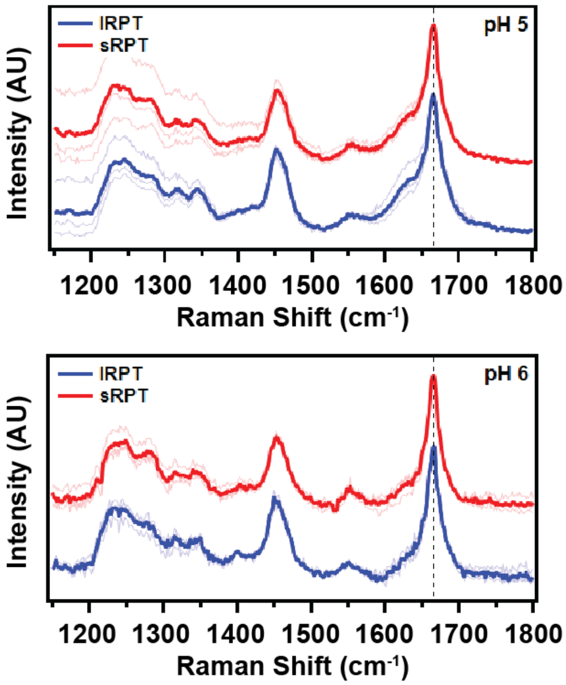

Fig. S1. lRPT and sRPT fibrils form similar, β-sheet-rich, secondary structure. lRPT (blue) and sRPT (red) fibrils were analyzed by Raman spectroscopy. Spectra were collected at multiple spatial locations (thin lines), which were then averaged (bold line). The vertical dashed line represents 1665 cm^{-1}, to which data were normalized. sRPT spectra are off-set for clarity.

Figure S2

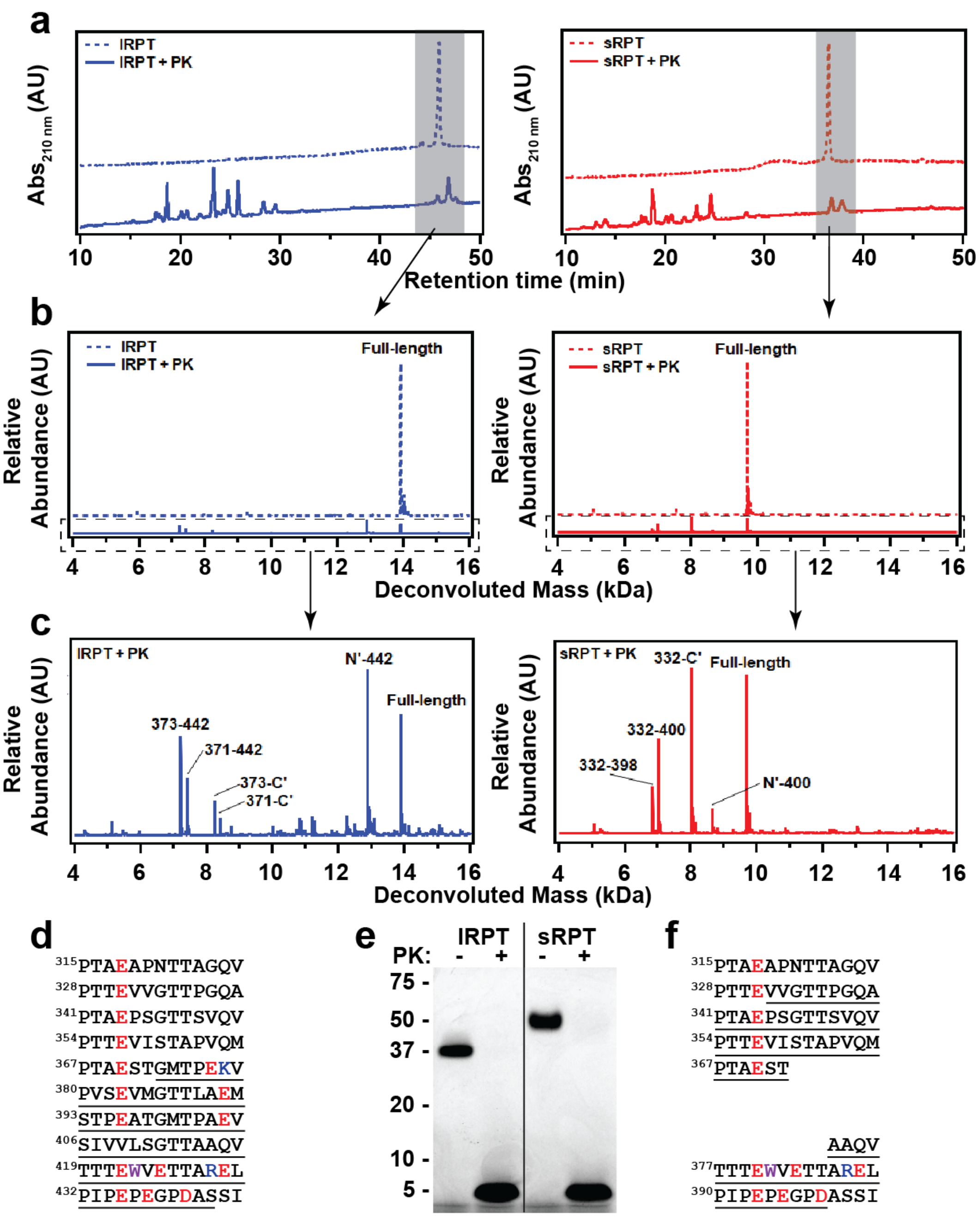

Fig. S2. Limited protease digestion of lRPT and sRPT. Unseeded lRPT (blue) or sRPT (red) fibrils (30 µM, formed at pH 6) were incubated in the absence (dashed) or presence (solid) of 0.3 µg/mL PK at 37 °C for 15 h and analyzed by LC-MS (**a-c**) and SDS-PAGE (**e**). Peaks corresponding to full-length/large peptides (shaded areas in **a**) were deconvoluted to identify the singly-charged masses (**b-c**). Fragments were mapped to the lRPT (**d**) or sRPT (**f**) sequence. The underlined regions represent the smallest protease-resistant fragment identified in (**c**). Data are representative of 2 independent experiments. The paradoxical migration of full-length lRPT at ~37 kDa and sRPT at ~50 kDa has been shown prior (McGlinchey et al. 2011 *J. Biol. Chem.*).

Figure S3

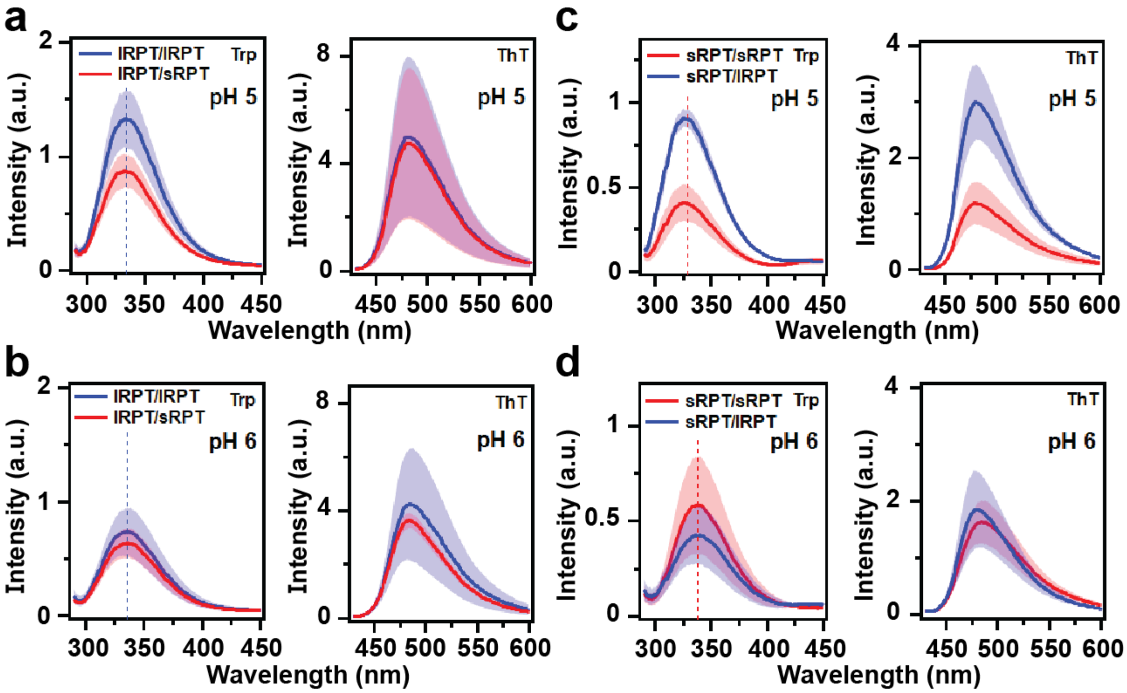

Fig. S3. Trp and ThT fluorescence of self- and cross-seeded RPT fibrils. lRPT (**a–b**) and sRPT (**c–d**) self- and cross-seeded fibrils, grown at pH 5 or 6, respectively. Spectra are normalized for respective concentration. Vertical dashed lines represent λ_{max} for unseeded lRPT (**a–b**) or sRPT (**c–d**) for comparison. Solid line and shaded area represent the mean and standard deviation, respectively, from 3 independent experiments.

Figure S4

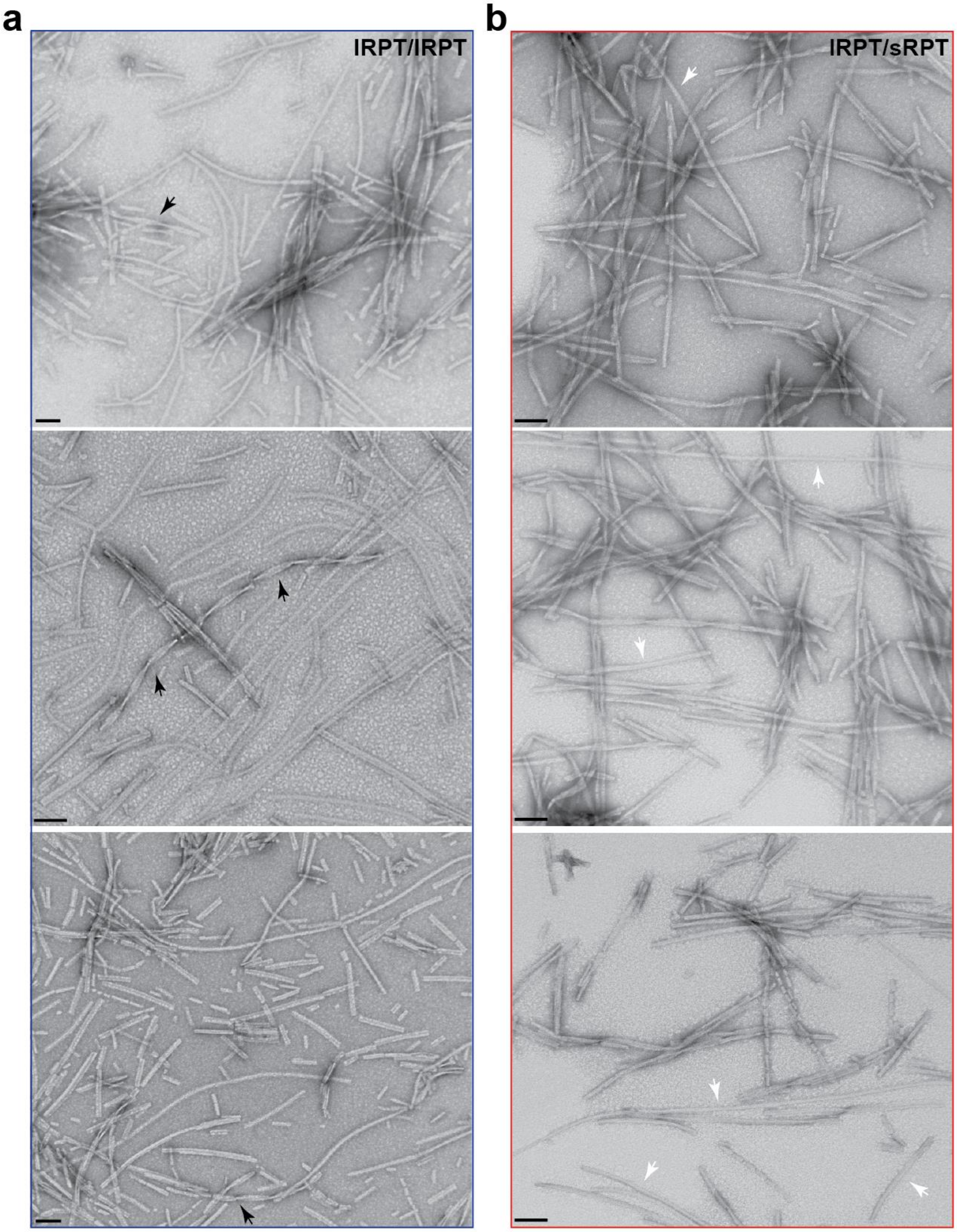

Fig. S4. Additional TEM images of lRPT/lRPT (**a**) and lRPT/sRPT (**b**) fibrils formed at pH 6. Black and white arrows represent twisted and straight filaments, respectively. Scale bars indicate 100 nm.

Figure S5

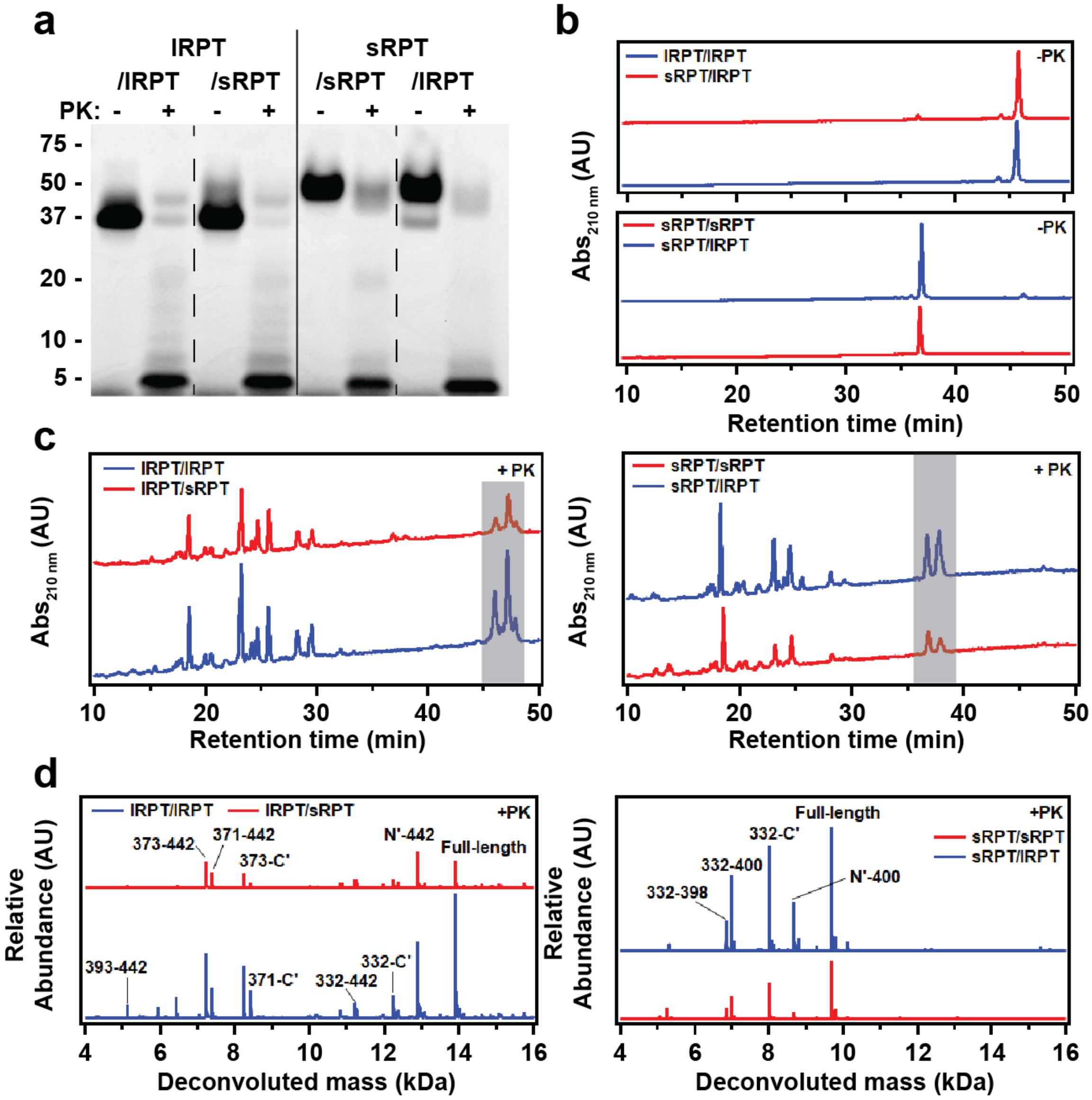

Fig. S5. Cross-seeding does not alter the protease-assessible regions of RPT at pH 6. Self- and cross-seeded fibrils (30 µM, formed at pH 6), were incubated in the absence and presence of 0.3 µg/mL PK at 37 ˚C for 8 h and analyzed by SDS-PAGE (**a**) and LC-MS (**b-d**). Peaks corresponding to full-length/large peptides (shaded areas in **c**) were deconvoluted to identify the singly-charged masses (**d**). Data are representative of 2 independent experiments.

Figure S6

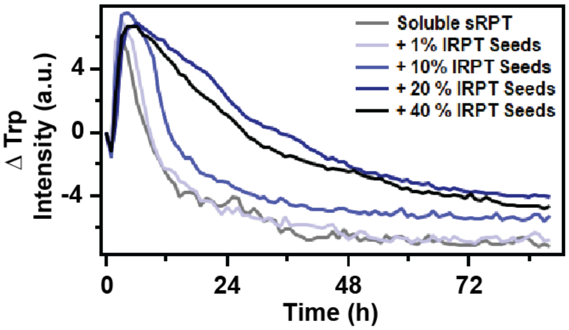

Fig. S6. lRPT fibrils inhibit the maturation phase of sRPT aggregation. The aggregation of 30 µM sRPT alone (grey) and in the presence of 0.3 (1%, light blue), 3 (10%, blue), 6 (20%, dark blue) or 12 µM (40%, black) lRPT seeds at pH 5. Reactions were monitored under constant shaking (6 mm) at 37 °C. Data represent an average of 4 replicates.

Figure S7

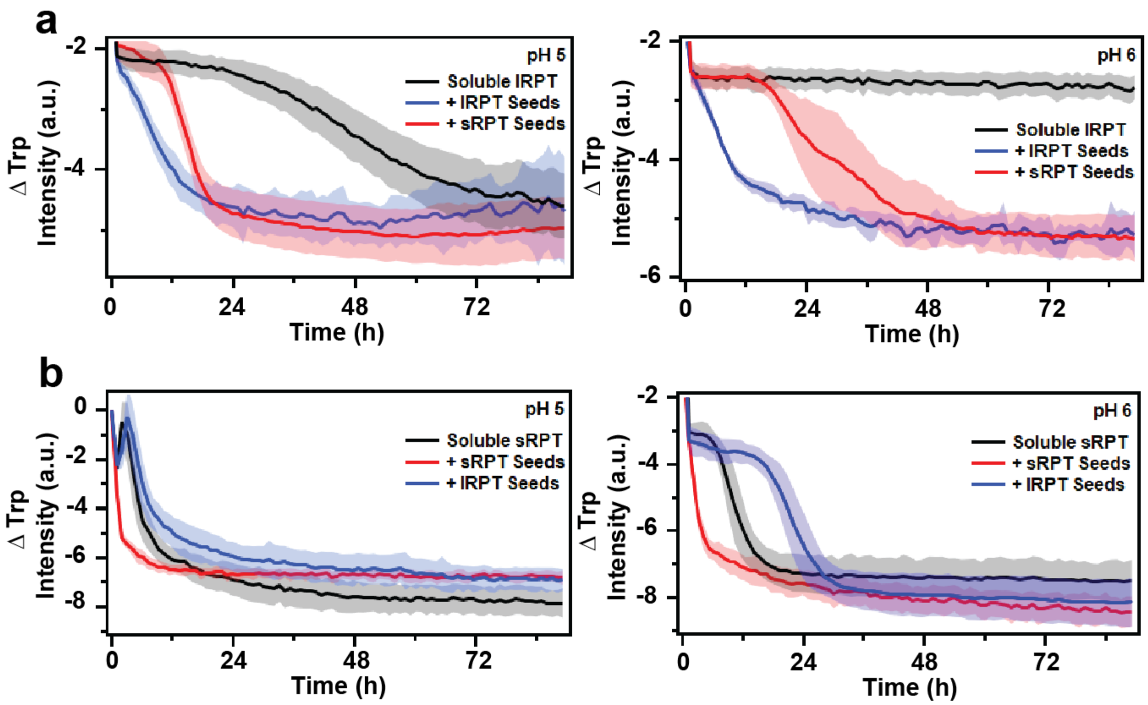

Fig. S7. Additional data set for self- and cross-seeding reactions. The aggregation of 30 µM lRPT (**a**) or sRPT (**b**) alone (black) and in the presence of 3 µM lRPT (blue) or sRPT (red) seeds at pH 5 and 6. Reactions were monitored under constant linear shaking (6 mm) at 37 °C. Solid line and shaded area represent the mean and standard deviation, respectively, from ≥5 replicates.

Figure S8

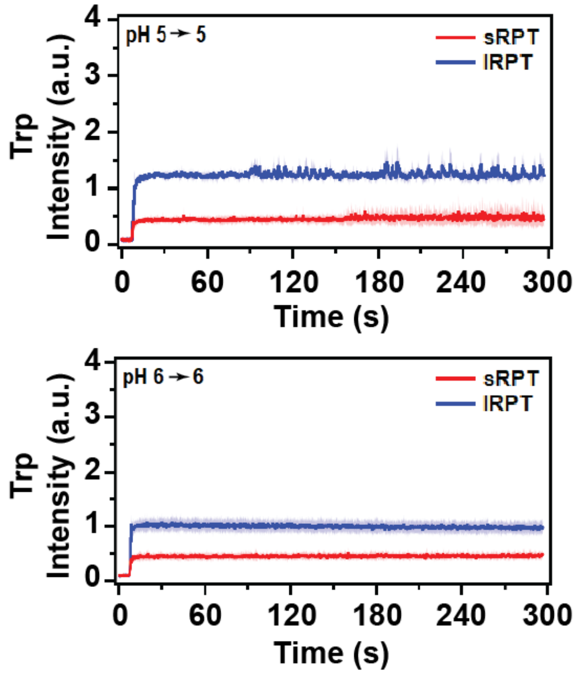

Fig. S8. Self-dilution does not result in disaggregation. lRPT (blue) and sRPT (red) fibrils (10 μM), formed at pH 5 or 6 were diluted 6-fold into a cuvette containing pH 5 or 6 buffer and Trp emission was monitored under constant stirring at 25 ˚C. The bold line and shaded area represent the mean and standard deviation from 3 independent experiments, respectively.

Figure S9

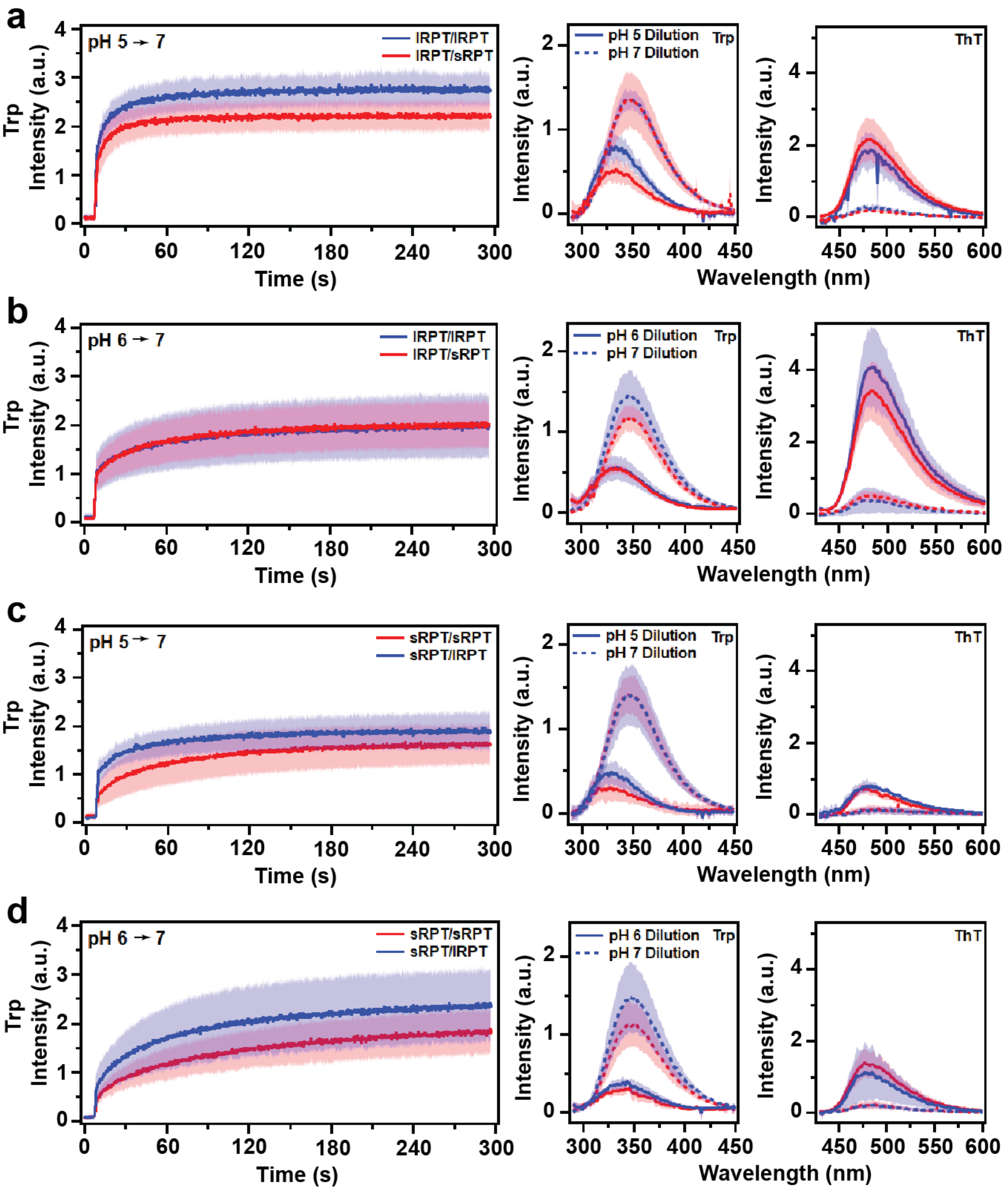

Fig S9. Disaggregation of self- and cross-seeded RPT fibrils. lRPT (**a–b**) and sRPT (**c–d**) self- and cross-seeded fibrils, grown at pH 5 or 6, were diluted 6-fold into pH 7 buffer and Trp emission was monitored under constant stirring at 25 °C. Trp and ThT fluorescence were collected after self-dilution (solid lines) or dilution into pH 7 buffer (dashed lines). Bold line and shaded area represent the mean and standard deviation from 3 independent experiments, respectively.
